# The *Caenorhabditis elegans* Iodotyrosine Deiodinase Ortholog SUP-18 Functions through a Conserved Channel SC-Box to Regulate the Muscle Two-Pore Domain Potassium Channel SUP-9

**DOI:** 10.1371/journal.pgen.1004175

**Published:** 2014-02-20

**Authors:** Ignacio Perez de la Cruz, Long Ma, H. Robert Horvitz

**Affiliations:** 1 Howard Hughes Medical Institute, Department of Biology, Massachusetts Institute of Technology, Cambridge, Massachusetts, United States of America; 2 State Key Laboratory of Medical Genetics, School of Life Sciences, Central South University, Changsha, Hunan, China; Texas A&M University, United States of America

## Abstract

Loss-of-function mutations in the *Caenorhabditis elegans* gene *sup-18* suppress the defects in muscle contraction conferred by a gain-of-function mutation in SUP-10, a presumptive regulatory subunit of the SUP-9 two-pore domain K^+^ channel associated with muscle membranes. We cloned *sup-18* and found that it encodes the *C. elegans* ortholog of mammalian iodotyrosine deiodinase (IYD), an NADH oxidase/flavin reductase that functions in iodine recycling and is important for the biosynthesis of thyroid hormones that regulate metabolism. The FMN-binding site of mammalian IYD is conserved in SUP-18, which appears to require catalytic activity to function. Genetic analyses suggest that SUP-10 can function with SUP-18 to activate SUP-9 through a pathway that is independent of the presumptive SUP-9 regulatory subunit UNC-93. We identified a novel evolutionarily conserved serine-cysteine-rich region in the C-terminal cytoplasmic domain of SUP-9 required for its specific activation by SUP-10 and SUP-18 but not by UNC-93. Since two-pore domain K^+^ channels regulate the resting membrane potentials of numerous cell types, we suggest that the SUP-18 IYD regulates the activity of the SUP-9 channel using NADH as a coenzyme and thus couples the metabolic state of muscle cells to muscle membrane excitability.

## Author Summary

Iodotyrosine deiodinase (IYD) controls the recycling of iodide in the biogenesis of thyroid hormones that regulate metabolism. Defects in IYD result in congenital hypothyroidism, a multisystem disorder that can lead to growth failure and severe mental retardation. We identified the gene *sup-18* of the nematode *Caenorhabditis elegans* as a regulator of the SUP-9/UNC-93/SUP-10 two-pore domain potassium channel complex and showed that SUP-18 is an ortholog of IYD, a member of the NADH oxidase/flavin reductase family. SUP-18 IYD is required for the activation of the channel complex by a gain-of-function mutation of the SUP-10 protein. SUP-9 channel activation by SUP-18 requires a conserved serine-cysteine-rich region in the C-terminus of SUP-9 and is independent of the function of the conserved multi-transmembrane protein UNC-93. We propose that SUP-18 uses NADH as a coenzyme to activate the SUP-9 channel in response to the activity of SUP-10 and the metabolic state of muscle cells.

## Introduction

Hypothyroidism, one of the most common endocrine disorders, can cause many different symptoms and can lead to defects in brain development and maturation and retarded postnatal development [1]. For thyroid hormone biosynthesis, iodide is recycled by iodotyrosine deiodinase through the deiodination of monoiodotyrosine and diiodotyrosine, two byproducts in the generation of thyroid hormones [2]–[6]. In humans, this deiodination is catalyzed by human iodotyrosine dehalogenase (DEHAL1)/iodotyrosine deiodinase (IYD), an NADH oxidase/flavin reductase [7]–[10]. Mutations in IYD cause congenital hypothyroidism [11]–[13]. How the activity of IYD is regulated *in vivo* and whether IYD has other functions remain to be elucidated.

Four transmembrane/two-pore domain K^+^ channels play a key role in establishing the resting membrane potentials of many cell types and in modulating their responses to neurotransmitters and second messengers [14]–[16]. To date, 15 human two-pore domain K^+^ channels have been identified [14], [16], [17]. The activities of two-pore domain K^+^ channels can be regulated by multiple chemical and physical factors, including temperature [18], membrane stretch [19], [20], arachidonic acid [21], pH [22], [23], volatile anesthetics [24], [25] and neurotransmitters [26], [27].

The gene *sup-9* of the nematode *Caenorhabditis elegans* encodes a two-pore domain K^+^ channel [28]. *sup-9(n1550)* gain-of-function (gf) mutants are egg-laying defective and display a flaccid paralysis and a rubberband uncoordinated (Unc) behavior: when prodded on the head, a *sup-9(n1550gf)* worm contracts and relaxes along its entire body without moving backwards, while a wild-type worm contracts its anterior end and moves away [29]. Loss-of-function (lf) mutations in *sup-9* or two other genes, *sup-10* and *unc-93*, completely suppress these *sup-9(n1550gf)* defects [29]–[31]. In addition, gf mutations in *sup-10* and *unc-93* themselves induce a rubberband Unc paralysis, which in turn are suppressed by lf mutations in *sup-9*, *sup-10* and *unc-93* [30]–[32]. lf mutants of *unc-93*, *sup-9* and *sup-10* do not have obviously abnormal phenotypes [29]–[31], [33]. The SUP-9 two-pore domain K^+^ channel is most closely related to human TASK-3 [28], [34], [35]. *unc-93* encodes a conserved multi-pass transmembrane protein [33]. An UNC-93 homolog, UNC93b1, is involved in innate immune responses in mammals [36], [37]. *sup-10* encodes a novel type-I transmembrane protein [35]. Genetic analyses and the molecular identities of these genes suggest that *in vivo* SUP-10 and UNC-93 form a protein complex with the SUP-9 two-pore domain K^+^ channel and modulate its activity as regulatory subunits [28], [33].

Mutations in the gene *sup-18* suppress the muscle defects caused by gf mutations in these three genes, strongly suppressing the locomotory defects of *sup-10(n983gf)* mutants, partially suppressing the locomotory defects of the strong *unc-93(e1500gf)* mutants, the weak *unc-93(n200gf)* mutants and the strong *sup-9(n1550gf)/+* heterozygous mutants, and suppressing only the lethality of *sup-9(n1550gf)* mutants [29], [30] (also see Table 1 below). In this study we report that *sup-18* encodes the *C. elegans* ortholog of mammalian iodotyrosine deiodinase/dehalogenase (IYD) [7], [8], [10]. Our findings suggest that SUP-18 is a functional regulator of the SUP-9/SUP-10/UNC-93 two-pore domain K^+^ channel complex *in vivo* and that IYD might function with two-pore domain K^+^ channel complexes in mammals.

**Table 1:** *sup-18(lf)* mutations specifically suppress *sup-10(n983gf)* locomotory defects.

Genotype	LR ± SEM	n
Wild-type	27.3±0.5	36
*sup-9(n1550gf)* *	Inviable	
*sup-9(n1550gf)/+*	1.3±0.4	16
*sup-10(n983gf)*	5.1±0.5	16
*unc-93(e1500gf)*	0.2±0.1	32
*unc-93(n200gf)*	16.1±0.5	24
*sup-18(n1030)*	25.3±0.7	16
*sup-9(n1550gf); sup-18(n1030)*	0.0±0.0	12
*sup-9(n1550gf)/+; sup-18(n1030)*	4.6±0.5	16
*unc-93(e1500gf) sup-18(n1030)*	1.0±0.3	35
*unc-93(n200gf) sup-18(n1030)*	21.4±0.5	36
*sup-18(n1030); sup-10(n983gf)*	26.4±0.6	16
*sup-9(n1913); unc-93(e1500gf)*	27.1±0.5	11
*unc-93(e1500gf); sup-10(n4025)*	13.9±0.6	30
*unc-93(e1500gf) sup-18(n1030); sup-10(n4025)*	19.1±0.4	30
*unc-93(e1500gf); sup-10(n4026)*	14.9±0.5	30
*unc-93(e1500gf) sup-18(n1030); sup-10(n4026)*	19.6±0.4	30

Young adult hermaphrodites were assayed for the number of body bends made on a bacterial lawn in 1 min interval.

* : *sup-9(n1550gf)* homozygous animals are inviable [35].

LR: locomotory rate (bodybends/min).

## Results

### *sup-18* has gene-specific effects on the rubberband Unc phenotype

*sup-10(n983gf)* mutants have a reduced locomotory rate (Table 1). A loss-of-function mutation in *sup-18*, *n1030*, restores wild-type locomotion to *sup-10(n983gf)* mutants (Table 1) [30]. *unc-93(n200gf)* causes a less severe rubberband Unc phenotype than *sup-10(n983gf)*, yet the *unc-93(n200gf)* phenotype is still only partially suppressed by *sup-18(n1030)* (Table 1). *unc-93(e1500gf)* mutants, which have a more severe rubberband Unc phenotype than *sup-10(n983gf)* mutants, similarly are only weakly suppressed by *sup-18(n1030)*. These results suggest that the differential suppression of the rubberband Unc mutants by *sup-18(n1030)* is caused by gene-specific effects rather than by differential severity of paralysis in these mutants.

We further tested this notion using weakly paralyzed double mutants carrying the *unc-93(e1500gf)* mutation and a partial lf allele of *sup-10*. Introduction of the *sup-18(n1030)* mutation into partially suppressed *unc-93(e1500gf); sup-10(n4025)* or *unc-93(e1500gf); sup-10(n4026)* mutants only weakly improved their locomotory rates from approximately 14 to 19 body-bends/minute (Table 1). These results confirm that *sup-18(n1030)* only weakly suppresses gf mutations in *unc-93*.

The suppression of *sup-10(n983gf)* depends on the dosage of the *sup-18* allele [29]. We found that *sup-18(n1030)/+; sup-10(n983gf)* males exhibit an intermediate phenotype (15.2 bends/min) between those of the more severely paralyzed *sup-10(n983gf)* males (4.7 bends/min) and the strongly suppressed *sup-18(n1030); sup-10(n983gf)* males (31.7 bends/min) (Table 2). This dose-dependent effect was observed for all lf alleles of *sup-18* tested (Table 2). By contrast, the suppression of *sup-10(n983gf)* by *sup-9(n1913)*, a channel null allele, was recessive.

**Table 2:** *sup-18(lf)* mutations exhibit dosage-dependent suppression of the locomotory defects of *sup-10(n983gf)* mutant.

Genotype	LR ± SEM	n
Wild-type	33.0±1.2	36
*sup-10(n983gf)*	4.7±0.9	25
*sup-18(n1030); sup-10(n983gf)*	31.7±0.7	15
*sup-18(n1030)/+; sup-10(n983gf)*	15.2±0.7	25
*sup-18(n1033)/+; sup-10(n983gf)*	13.8±0.6	25
*sup-18(n1014)/+; sup-10(n983gf)*	14.6±0.6	25
*sup-18(n1036)/+; sup-10(n983gf)*	12.3±0.7	25
*sup-18(n1471)/+; sup-10(n983gf)*	14.0±0.7	25
*sup-9(n1913); sup-10(n983gf)*	33.2±1.1	15
*sup-9(n1913)/+; sup-10(n983gf)*	5.6±0.6	20
*unc-93(e1500gf); sup-10(n4025)*	10.0±0.5	30
*unc-93(e1500gf) sup-18(n1030)/+; sup-10(n4025)*	9.8±0.7	20
*unc-93(e1500gf) sup-18(n1030); sup-10(n4025)*	17.5±0.9	30
*sup-9(n1550gf)/+*	3.8±0.5	24
*sup-9(n1550gf)/+; sup-18(n1030)/+*	5.0±0.5	39
*sup-9(n1550gf)/+; sup-18(n1030)*	9.8±0.5	22

Young adult males were assayed for the number of bends made on a bacterial lawn during 1 min interval. LR: locomotory rate (bodybends/min).

Because the weak suppression of the locomotory defect of *unc-93(e1500gf)* mutants by *sup-18(lf)* mutations (Table 1) [30] makes a dosage analysis of *sup-18(lf)* suppression of *unc-93(e1500gf)* difficult, we examined weakly paralyzed *unc-93(e1500gf); sup-10(n4025)* males, which are more visibly suppressed by *sup-18(n1030)* (Table 2). We found that the locomotory rate of *unc-93(e1500gf); sup-10(n4025)* males heterozygous for *sup-18(n1030)* was similar to that of males wild-type for *sup-18* (10.0 vs. 9.8, respectively) (Table 2). Similarly, *sup-9(n1550gf)/+; sup-18(n1030)/+* males had only slightly improved locomotion compared to *sup-9(n1550gf)/+* males (5.0 vs. 3.8, respectively) (Table 2). We conclude that the dose-dependent suppression of rubberband Unc mutants by *sup-18* alleles is also gene-specific: the *sup-10(n983gf)* phenotype is much more sensitive to *sup-18* levels than is that of the other rubberband mutants.

### *sup-18* encodes the *C. elegans* ortholog of mammalian iodotyrosine deiodinase

**Figure 1:**
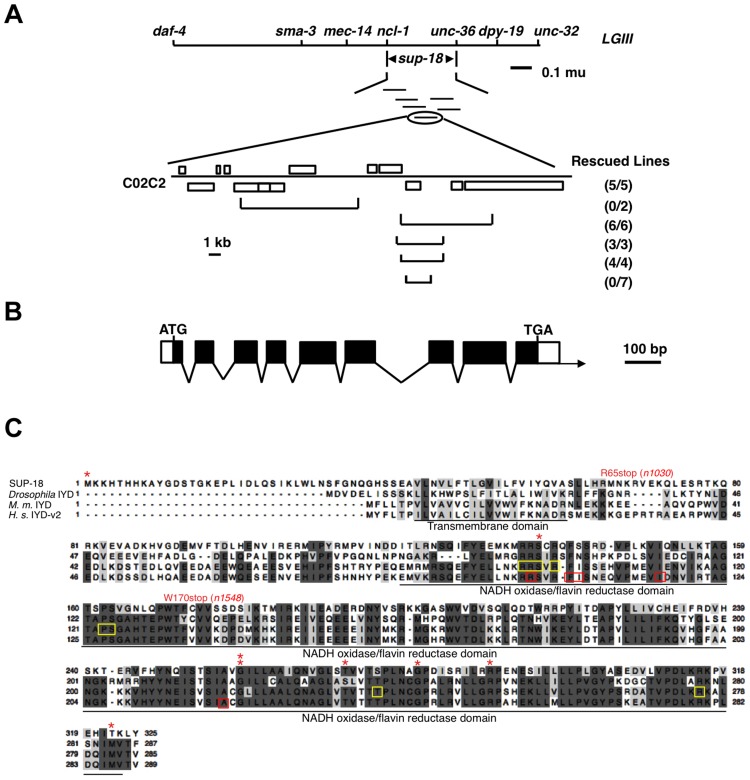
*sup-18* encodes the *C. elegans* ortholog of mammalian iodotyrosine deiodinase. (A) Top, genetic map of the *sup-18* region of linkage group III (LG III). Horizontal lines below *sup-18* represent the cosmids tested for rescue of *sup-18(n1010); sup-10(n983gf)* mutants. Bottom, physical map of cosmid C02C2. Open boxes, coding regions; horizontal brackets, cosmid subclones. Rescued lines, number of independently derived transgenic lines/total number of lines scored. Rescue was scored as the appearance of animals with the phenotype of rubberband Unc paralysis. (B) Intron-exon structure of *sup-18* as inferred by comparison of the cDNA and genomic sequences. Dark boxes, coding regions; open boxes, untranslated regions; arrow, direction of transcription. The *sup-18* open reading frame is 978 bp, the 5′ UTR is 57 bp and the 3′UTR is 44 bp. (C) Sequence alignment of SUP-18 and iodotyrosine deiodinases from other species. Amino acids conserved in at least three species are darkly shaded, while amino acids with similar physical properties in at least three species are lightly colored. *: *sup-18* missense mutations (see Table 3). Residues mutated in human hypothyroidism patients are indicated by red boxes. The FMN cofactor binding residues in mouse IYD are indicated by yellow boxes. The transmembrane domain and NADH oxidase/flavin reductase domain are underlined and labeled. Genebank accession numbers are as follows: SUP-18, JX978835; *Drosophila*, AAM11009; *M. musculus*, AAH23358; *H. sapien*s, NP_981932.

*sup-18* had previously been mapped to the interval between *daf-4* and *unc-32* on LGIII [30]. Using three-point mapping we further localized *sup-18* to the interval between *ncl-1* and *unc-36* (see Materials and Methods) (Figure 1A). Transgene rescue experiments with cosmids spanning the *ncl-1*-to-*unc-36* interval and with smaller cosmid subclones identified a 4.5 kb minimal rescuing fragment from cosmid C02C2: as a transgene, this fragment restored the rubberband Unc phenotype to *sup-18(n1010); sup-10(n983gf)* mutants (Figure 1A). This rescuing fragment contained a single predicted gene, *C02C2.5* [www.wormbase.org]. We screened a mixed-stage cDNA library [38] using the smallest cosmid subclone with *sup-18* rescuing activity and obtained a single partial cDNA of this predicted gene. We defined the structure of this gene from RT-PCR and RACE experiments (see Materials and Methods) (Figure 1B).

*sup-18* encodes a predicted protein of 325 amino acids. This protein is the only *C. elegans* ortholog of mammalian iodotyrosine deiodinase (IYD), which belongs to the NADH oxidase/flavin reductase superfamily (Fig. 1C) [7]–[10]. IYD catalyzes the recycling of iodide by deiodinating 3′-monoiodotyrosine and 3′, 5′-diiodotyrosine, the main byproducts in the process of thyroid hormone biogenesis [2]–[5], [7], [8]. The identity between SUP-18 and human IYD protein variant 2 (also named DEHAL1) [8] is 31% overall and 45% over the NADH oxidase/flavin reductase domain (Figure 1C). Like IYDs of *Drosophila*, mouse and human, SUP-18 has a hydrophobic region that precedes the NADH oxidase/flavin reductase domain and might serve as a transmembrane domain.

We identified molecular lesions in the *sup-18* coding sequence of all 18 mutant strains analyzed (Table 3, Fig. 1C). The *sup-18(n1033)* mutation leads to the substitution of an isoleucine for the initiator methionine, which should cause any translational products to be nonfunctional. (The next three ATG sequences in the *sup-18* cDNA are out of frame.) The *sup-18(n1030)* and *sup-18(n1548)* mutations cause premature stop codons that likely generate truncated protein products. Four mutations (*n1038*, *n527*, *n463*, *n1539*) cause a frameshift. Another four mutations (*n1036*, *n1035*, *n1015*, *n1558*) affect splice donor or acceptor sites. The remaining seven missense mutations (*n1010*, *n1554*, *n1471*, *n1556*, *n1014*, *n1022*, *n528*) disrupt residues within the NADH oxidase/flavin reductase domain.

**Table 3:** *sup-18* loss-of-function mutations.

Allele	Mutation	Effect	Mutagen	Background
*n1033*	ATG to ATT	M1I	EMS	*sup-10(n983gf)*
*n1030*	CGA to TGA	R65stop	EMS	*sup-10(n983gf)*
*n1038*	916 bp deletion	97+frameshift	EMS	*unc-93(e1500gf)*
*n527*	13 bp deletion	154+frameshift	Spont	*unc-93(e1500gf)*
*n1548*	TGG to TAG	W170stop	EMS	*sup-10(n983gf)*
*n463*	4 bp deletion	175+frameshift	Spont	*unc-93(e1500gf)*
*n1539*	Tc3 Insertion	320+frameshift	Spont	*sup-10(n983gf)*
*n1036*	agGT to aaGT	3^rd^ splice acceptor	EMS	*sup-10(n983gf)*
*n1035*	agGC to aaGC	5^th^ splice acceptor	EMS	*sup-10(n983gf)*
*n1015*	GTgt to GTat	8^th^ splice donor	EMS	*sup-10(n983gf)*
*n1558*	agAT to aaAT	8^th^ splice acceptor	EMS	*sup-10(n983gf)*
*n1010*	AGT to AAT	S137N	EMS	*sup-10(n983gf)*
*n1554*	GGC to AGC	G258D	EMS	*sup-10(n983gf)*
*n1471*	GGC to GAC	G258S	Gamma	*sup-10(n983gf)*
*n1556*	ACT to ATT	T271I	EMS	*sup-10(n983gf)*
*n1014*	GGA to AGA	G280R	EMS	*sup-10(n983gf)*
*n1022*	AGG to AAG	R289K	EMS	*sup-10(n983gf)*
*n528*	ACC to CCC	T322P	Spont	*unc-93(e1500gf)*

DNA sequences were determined for both strands of *sup-18* exons and intron/exon boundaries of each mutant. For splice-junction mutants, the intron sequence is indicated by lowercase and the exon sequence by uppercase letters. EMS, ethyl methanesulfonate; Spont, spontaneous. Frameshift, mutations causing frameshift after the indicated codons.

### SUP-18 and SUP-10 are similarly localized within muscles

**Figure 2:**
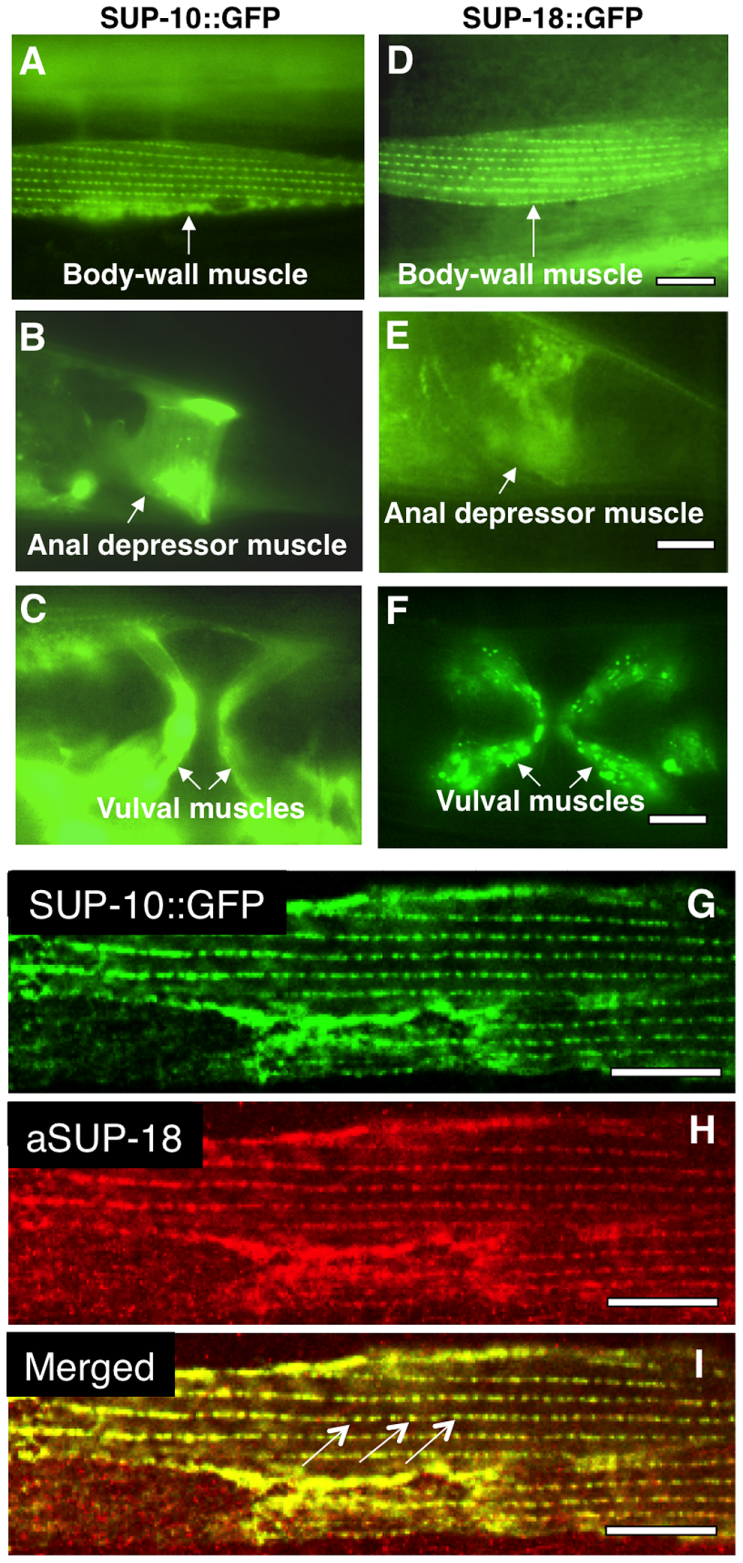
SUP-18 is expressed predominantly in muscles and co-localizes subcellularly with SUP-10. Epifluorescence images of worms carrying (A–C) a P*sup-10::gfp* translational fusion transgene or (D–F) a P*sup-18::gfp* translational fusion transgene. (A, D) Body-wall muscle cells displaying GFP fluorescence in dense body-like structures. (B, E) Tail regions of transgenic animals showing fluorescence in the anal depressor muscles (arrows). (C, F) Ventral views of transgenic animals showing fluorescence in vulval muscles (arrows). (G, H, I) Confocal microscopic images of an animal expressing a P*sup-10::gfp* translational fusion transgene and a P*_myo-3_sup-18* transgene. SUP-10::GFP fusion protein (G) was visualized by GFP signals and SUP-18 (H) was detected by immunostaining with a rabbit anti-SUP-18 polyclonal antibody (see Materials and Methods). (I) The merged picture indicates colocalization of SUP-10::GFP and SUP-18 in the dense bodies (arrows). Scale bars, 10 µm.

To examine the expression pattern of *sup-18*, we introduced the coding sequence of *gfp* between codons 88 and 89 of a genomic clone of *sup-18*, generating a *sup-18* translation fusion transgene (see Materials and Methods). Similar to transgenic animals expressing a P*sup-10::gfp* translational fusion transgene, P*sup-18::gfp* transgenic animals displayed GFP fluorescence in body-wall (Fig. 2A, D), defecation (Fig. 2B, E) and vulval muscles (Fig. 2C, F). In body-wall muscle cells (Fig. 2A, D), the SUP-10::GFP and SUP-18::GFP fusion proteins both localized to cell-surface regions aligned with dense bodies, the functional analogs to vertebrate Z-lines that connect the myofibril lattice to the cell membrane [39]. In addition to muscles, three neurons in the head of P*sup-18::gfp* transgenic animals also displayed GFP staining (I. de la Cruz and H. R. Horvitz, unpublished observations). We previously reported expression of a P*sup-9::gfp* reporter in the four SIA interneurons [28]. We stained the P*sup-18::gfp* transgenic animals with an anti-CEH-17 antibody, which labels the four SIA neurons and the ALA neuron [40], and found that the neurons expressing the SUP-18::GFP fusion protein were not the SIAs (I. de la Cruz and H. R. Horvitz, unpublished observations).

We generated a rabbit anti-SUP-18 antibody (see Materials and Methods). In immunostained animals, this antibody could detect overexpressed SUP-18 but failed to detect endogenous SUP-18, probably because of the low level of SUP-18 expression. We next generated transgenic animals co-expressing a P*sup-10::gfp* fusion transgene and *sup-18* under control of a *myo-3* promoter [41] and examined the subcellular expression of SUP-18 using the antibody and of SUP-10::GFP using GFP fluorescence. We found that SUP-10 and SUP-18 colocalize in subcellular structures, including the dense bodies in the body-wall muscles (Fig. 2G, H, I). Since GFP fusions to SUP-9 and UNC-93 localize similarly [28], this result suggests that SUP-18 colocalizes with a SUP-9/UNC-93/SUP-10 complex.

### SUP-18 is a type-I transmembrane protein that can function independently of membrane anchoring

**Figure 3:**
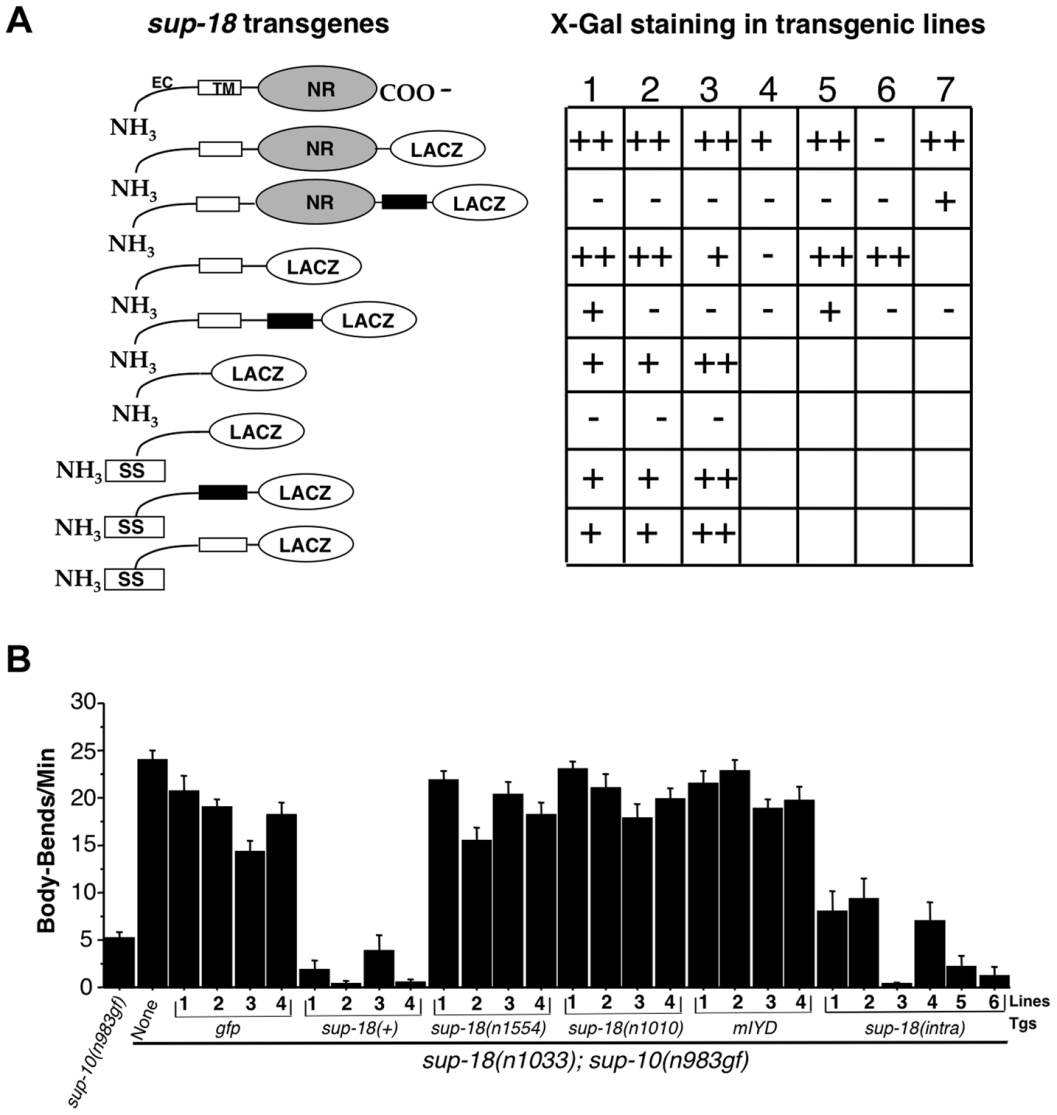
SUP-18 is a type-I transmembrane protein with an NADH oxidase/flavin reductase domain that resides intracellularly and can function without plasma membrane localization. (A) Transgenes expressing a SUP-18::β-galactosidase fusion protein were transformed into wild-type animals using the dominant *rol-6* coinjection marker. *β-*galactosidase assays were performed as described [77]. At least 100 animals per transgenic line were scored for lacZ staining using Nomarski optics. Quantification was as following: staining of adult vulval muscles and larvae easily observed at 10× magnification in >30% of animals (++); staining of 3–30% of animals (+); staining of fewer than 3% of animals (−); no transgenic lines, not labeled. Schematic: open boxes, SUP-18 transmembrane domain; black boxes, a synthetic transmembrane domain; EC, SUP-18 extracellular domain; SS, signal sequence; NR, NADH oxidase/flavin reductase domain. (B) Lines of *sup-18(n1033); sup-10(n983gf)* animals carrying transgenes (Tgs) driven by the *myo-3* promoter, as indicated, were scored for locomotory rates. P*_myo-3_ gfp* (pPD93.97) was used as a coinjection marker to allow the identification of transgenic animals by their GFP fluorescence. Non-transgenic *sup-10(n983gf)* and *sup-10(n1033); sup-10(n983gf)* mutants were scored as controls. Error bars, mean ± SEM; n = 12 for each transgenic line or control genotype. *sup-18(intra)*: transgenes expressing the SUP-18 intracellular domain (amino acid 66–325) fused with GFP.

Mammalian IYD is a transmembrane protein [7], [8]. The presence of a possible transmembrane domain in the predicted SUP-18 protein sequence (Fig. 1C) suggests that SUP-18 is also a transmembrane protein. To distinguish whether the NADH oxidase/flavin reductase domain of SUP-18 resides intracellularly or extracellularly, we generated transgenic animals expressing different SUP-18::β-galactosidase fusion proteins and assayed β-galactosidase activity *in vivo* in fixed animals (Fig. 3A). When β-galactosidase is localized intracellularly it is enzymatically active, whereas extracellular localization results in loss of β-galactosidase activity [42], [43]. The use of β-galactosidase activity to elucidate the membrane topology of *C. elegans* proteins *in vivo* has been reported previously for the presenilin SEL-12 protein [44] and for the MEC-4 sodium channel subunit [45].

Fixed transgenic animals expressing β-galactosidase fused to either the C-terminal region of SUP-18 or immediately C-terminal to the putative transmembrane domain showed robust β-galactosidase activity (Fig. 3A). Introduction of a synthetic transmembrane domain [45] between SUP-18 and β-galactosidase in these chimeras eliminated β-galactosidase enzymatic activity, presumably because the membrane orientation of β-galactosidase had been reversed (Fig. 3A).

These results strongly suggest that SUP-18 is a transmembrane protein and that the NADH oxidase/flavin reductase domain of SUP-18 resides intracellularly. But they do not distinguish between a type-I transmembrane protein (single-pass transmembrane protein with the N-terminal domain located extracellularly) and a cytoplasmic protein that simply localizes at the cell surface, *e.g.*, by interacting with another membrane protein or by linking to a GPI anchor [46]. To test if the putative transmembrane domain of SUP-18 can indeed behave as a transmembrane domain, we inserted a signal sequence at the N-terminus of SUP-18 (see Materials and Methods). While a fusion containing the presumptive extracellular domain of SUP-18 but lacking the putative transmembrane domain resided intracellularly as expected, the introduction of a signal sequence led to its secretion and loss of β-galactosidase enzymatic activity (Fig. 3A). By contrast, when either the SUP-18 putative transmembrane domain or the synthetic transmembrane domain [45] was added to this SUP-18::β-galactosidase fusion, the enzymatic activity was restored. These results indicate that the putative transmembrane domain of SUP-18 can indeed function as a transmembrane domain and suggest that SUP-18 is likely a type-I integral membrane protein, like IYD.

To establish an assay for *in vivo* SUP-18 activity, we expressed the *sup-18* coding sequence under the control of the *myo-3* promoter [41] in *sup-18(n1033); sup-10(n983)* mutant animals. While *sup-10(n983gf)* mutant animals are defective in locomotion, double mutants carrying the *sup-18(n1033)* null mutation had improved locomotory rates (Fig. 3B). Expression of P*_myo-3_ gfp* in *sup-18(n1033); sup-10(n983gf)* animals had little effect on their locomotory rate, whereas expression of a P*_myo-3_ sup-18(+)* transgene restored *sup-10(n983gf)* paralysis (Fig. 3B). By contrast, expression of two P*_myo-3_ sup-18* mutant constructs containing either the *n1554* missense mutation or the *n1010* mutation (which affects a conserved amino acid in the NADH oxidase/flavin reductase domain; Fig. 1C and Table 3) did not restore the rubberband Unc phenotype to *sup-18(n1033); sup-10(n983gf)* mutants (Fig. 3B).

We found that the mouse *IYD* gene could not substitute for *sup-18 in vivo* in restoring the rubberband Unc phenotype of *sup-18(n1033); sup-10(n983gf)* animals (Figure 3B). We tagged mouse IYD with GFP at its C-terminus and found that *C. elegans* expressing the fusion protein displayed GFP fluorescence in body-wall muscle structures similar to that observed for the SUP-18::GFP fusion (I. de la Cruz and H. R. Horvitz, unpublished observations). These results suggest that mouse IYD had been expressed properly and that mouse IYD might be inactive or otherwise incapable of substituting for SUP-18 in *C. elegans*.

Interestingly, transgenic expression of the SUP-18 intracellular domain alone (amino acids 66–325) was sufficient to restore rubberband Unc paralysis to *sup-18(n1033); sup-10(n983gf)* animals, although the rescue was less robust than that conferred by full-length SUP-18 (Fig. 3B). This finding suggests that the extracellular and transmembrane domains of SUP-18 are not essential for its *in vivo* function and is consistent with the conclusion that the NADH oxidase/flavin reductase domain is intracellular.

### Increased *sup-18(+)* expression in body-wall muscles specifically enhances the behavioral defects of *sup-10(n983gf)* mutants

The overexpression of *sup-18(+)* from a P*_myo-3_ sup-18(+)* transgene in *sup-18(n1033): sup-10(n983gf)* mutants not only restored the rubberband Unc phenotype but also apparently enhanced that phenotype beyond that of *sup-10(n983gf)* single mutants (Fig. 3B). This finding indicates a dose-dependent effect of *sup-18(+)* and is consistent with our gene-dosage observation that *sup-18(lf)/+* can partially improve the locomotory rate of *sup-10(n983gf)* mutants (Table 2). Overexpression of *sup-18(+)* with the coinjection marker *lin-15(+)* in *lin-15* mutant animals did not cause obvious differences in locomotion compared to animals injected with *lin-15(+)* alone (Table 4), indicating that overexpression of *sup-18(+)* itself did not slow locomotion.

**Table 4:** Overexpression of *sup-18* in body-wall muscles enhances the defects of *sup-10(n983gf)* but not *unc-93(e1500gf)* mutants.

Genotype	LR ± SEM	Brood size
Wild-type	26.8±0.4	ND
*lin-15; nEx[lin-15(+)#1]*	26.5±0.9	ND
*lin-15; nEx[lin-15(+)#2]*	27.3±0.7	ND
*lin-15; nEx[lin-15(+); sup-18(+)#1]*	27.1±0.8	ND
*lin-15; nEx[lin-15(+); sup-18(+)#2]*	26.9±0.8	ND
*lin-15; nEx[lin-15(+); sup-10(+); sup-18(+)#1]*	24.5±0.8	ND
*lin-15; nEx[lin-15(+); sup-10(+); sup-18(+)#2]*	25.2±0.8	ND
*sup-10(n983gf) lin-15; nEx[lin-15(+)#1]*	5.7±0.4	74±5
*sup-10(n983gf) lin-15; nEx[lin-15(+)#2]*	5.4±0.4	75±4
*sup-10(n983gf) lin-15; nEx[lin-15(+); sup-18(+)#1]*	0.1±0.1	27±3
*sup-10(n983gf) lin-15; nEx[lin-15(+); sup-18(+)#2]*	0.0±0.0	17±3
*sup-10(n983gf) lin-15; nEx[lin-15(+); sup-10(n983gf)#1]*	5.9±1.2	ND
*sup-10(n983gf) lin-15; nEx[lin-15(+); sup-10(n983gf)#2]*	6.6±0.8	ND
*sup-10(n983gf) lin-15; nEx[lin-15(+); sup-9(+)#1]*	6.0±0.6	ND
*sup-10(n983gf) lin-15; nEx[lin-15(+); sup-9(+)#2]*	5.1±0.4	ND
*sup-10(n983gf) lin-15; nEx[lin-15(+); unc-93(+)#1]*	5.1±0.3	ND
*sup-10(n983gf) lin-15; nEx[lin-15(+); unc-93(+)#2]*	5.8±0.5	ND
*unc-93(e1500gf); lin-15; nEx[lin-15(+)#1]*	0.0±0.0	35±2
*unc-93(e1500gf); lin-15; nEx[lin-15(+)#2]*	0.0±0.0	43±2
*unc-93(e1500gf); lin-15; nEx[lin-15(+); sup-18(+)#1]*	0.0±0.0	37±2
*unc-93(e1500gf); lin-15; nEx[lin-15(+); sup-18(+)#2]*	0.0±0.0	40±3
*sup-9(n1550gf); sup-18(n1030)*	0.0±0.0	24±1

Locomotion rate, mean for 12 animals. Brood size, mean for 10 animals. Four extrachromosomal arrays (*nEx*) were generated, two containing *lin-15(+)* alone and two containing both *lin-15(+)* and *sup-18(+)*, and were introduced into the different genetic backgrounds by mating to ensure consistent gene dosage among experiments. ND, not determined. LR: locomotory rate (bodybends/min).

**Figure 4:**
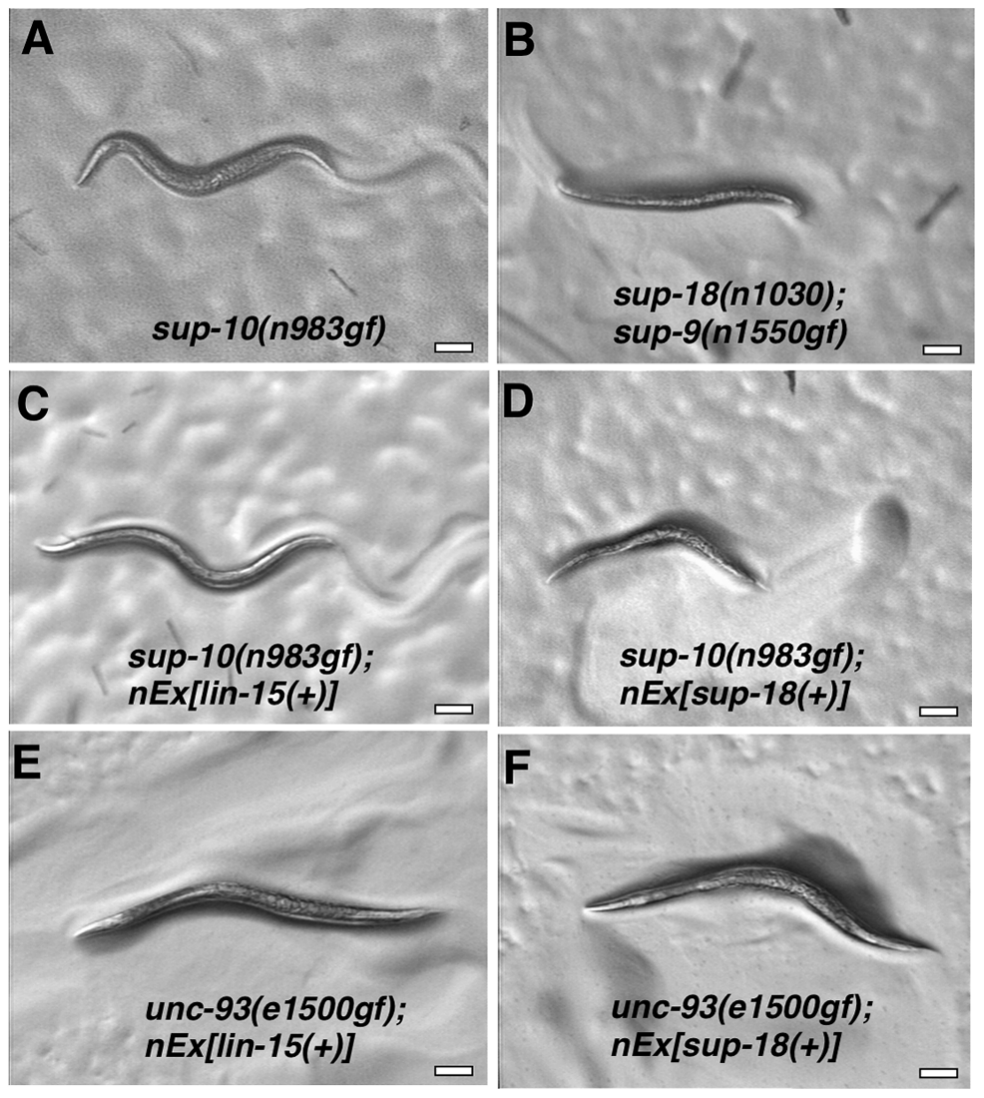
Overexpression of a *sup-18* transgene enhances the rubberband Unc phenotype of *sup-10(n983gf)* but not *unc-93(e1500gf)* mutants. Hermaphrodites were photographed at least 2 µm. Genotypes were as follows: (A) *sup-10(n983gf)*. (B) *sup-18(n1030); sup-9(n1550gf)*. (C) *sup-10(n983gf); lin-15(n765); nEx[lin-15(+)]*. (D) *sup-10(n983gf); lin-15(n765); nEx[lin-15(+)*, P*_myo-3_ sup-18(+)]*. (E) *unc-93(e1500gf); lin-15(n765); nEx[lin-15(+)]*. (F) *unc-93(e1500gf); lin-15(n765); nEx[lin-15(+)*, P*_myo-3_ sup-18(+)]*.

We introduced the extrachromosomal arrays containing the transgenes from two independently-derived strains carrying *sup-18(+)* and the *lin-15(+)* coinjection marker into *sup-10(n983gf) lin-15* double mutants by mating, so that each resulting strain would contain the same transgenes as the parental strain and therefore would overexpress *sup-18(+)* at equivalent levels. *sup-18(+)* overexpression caused a severe paralysis of *sup-10(n983gf) lin-15* animals relative to control transgenic animals expressing *lin-15* alone (0.1 and 0.0 vs. 5.7 and 5.4, bends/minute, respectively) (Table 4). *sup-10(n983gf)* mutants overexpressing *sup-18(+)* were smaller in size (Fig. 4A–D) and resembled severely paralyzed mutants carrying a *sup-9(n1550gf)* mutation (compare Figs. 4B and 4D).

We next tested if overexpression of *sup-9(+)*, *unc-93(+)* or *sup-10(n983gf)* itself could enhance the *sup-10(n983gf)* defect as did overexpression of *sup-18(+)*. Overexpression of these other genes under the control of the *myo-3* promoter did not affect the locomotory rate of transgenic *sup-10(n983gf)* mutant animals compared to animals transgenic for *lin-15* alone (Table 4). These results suggest that the activity of SUP-18 might be enhanced by increased expression, while increased expression of SUP-9, UNC-93 and SUP-10 does not increase the biological effects of these proteins.

We tested if overexpression of *sup-18(+)* could enhance the defects of *unc-93(e1500gf)* mutants and found no obvious difference in appearance compared to control animals overexpressing *lin-15* alone (Fig. 4E, F). Because the locomotory rate of *unc-93(e1500gf)* mutants transgenic for either *sup-18(+)* or *lin-15(+)* transgenes was zero (Table 4) and an enhancement of locomotory defects could not be scored, we turned to a different aspect of the phenotype of rubberband mutants, a reduced brood size [29]. Consistent with the enhancement of locomotory defects, overexpression of *sup-18(+)* reduced the brood size of *sup-10(n983gf)* mutants by three-fold, from an average of 74 and 75 progeny for the two transgenic lines, to 17 and 27, respectively (Table 4). These low brood sizes are comparable to those of severely paralyzed *sup-9(n1550gf); sup-18(n1030)* mutants (Table 4). By contrast, the brood sizes of *unc-93(e1500gf)* mutants did not change in response to *sup-18(+)* overexpression (35 and 43 vs. 37 and 40, respectively). Thus, the effects of *sup-18(+)* overexpression on the locomotion and brood size of rubberband Unc mutants are gene-specific: the *sup-10(n983gf)* phenotype is more sensitive to increased *sup-18* levels than is that of *unc-93(e1500gf)* mutants.

### The *sup-9(n1435)* mutation specifically suppresses the behavioral defects of *sup-10(n983gf)* mutants

Like *sup-18* mutations, the *sup-9* allele *n1435* strongly suppresses the locomotory defects of *sup-10(n983gf)* but not those of *unc-93(e1500gf)* mutants (Table 5) [29]. By contrast, null mutations in *sup-9*, such as *sup-9(n1913)*, completely suppress the defects caused by gf mutations in both *sup-10* and *unc-93* (Table 5) [30], [31]. To determine if other *sup-9* alleles exhibit similar gene-specific effects, we assayed 13 previously isolated *sup-9* missense mutations [34], [35], [36], [39]. Four *sup-9* mutations that had been isolated as *sup-10(n983gf)* suppressors and nine that had been isolated as *unc-93(e1500gf)* suppressors all strongly suppressed *unc-93(e1500gf)* and *sup-10(n983gf)* defects equally well (Table 5), confirming that *sup-9(n1435)* represents a rare class of *sup-9* mutations.

**Table 5:** Suppression of *sup-10(n983gf)* and *unc-93(e1500gf)* locomotory defects (bodybends) by *sup-9* mutations.

*sup-9* alleles	*sup-10(n983gf)*	*unc-93(e1500gf)*
Wild-type	5.1±0.5	0.1±0.1
*n1913* (null)	25.8±0.7	26.8±0.8
*n1435*	26.4±0.8	0.3±0.1
*n1016*	25.9±0.9	25.6±0.8
*n1025*	23.6±0.8	26.9±0.4
*n1472*	24.5±0.5	22.3±0.9
*n1557*	24.8±0.9	28.4±0.8
*lr35*	24.9±0.5	26.4±0.7
*lr45*	26.5±0.6	23.4±0.8
*lr100*	24.3±0.6	24.6±0.7
*lr129*	26.0±0.7	23.3±0.5
*lr142*	24.1±0.5	25.6±0.4
*n213*	27.2±0.7	24.2±0.5
*n264*	22.4±0.6	22.3±0.6
*n233*	26.1±0.6	26.7±0.7
*lr38*	26.7±0.5	26.4±0.6

Each assay represents the mean ± SEM of hermaphrodite bodybends assayed in 1 min intervals. n = 12 for all strains.

**Figure 5:**
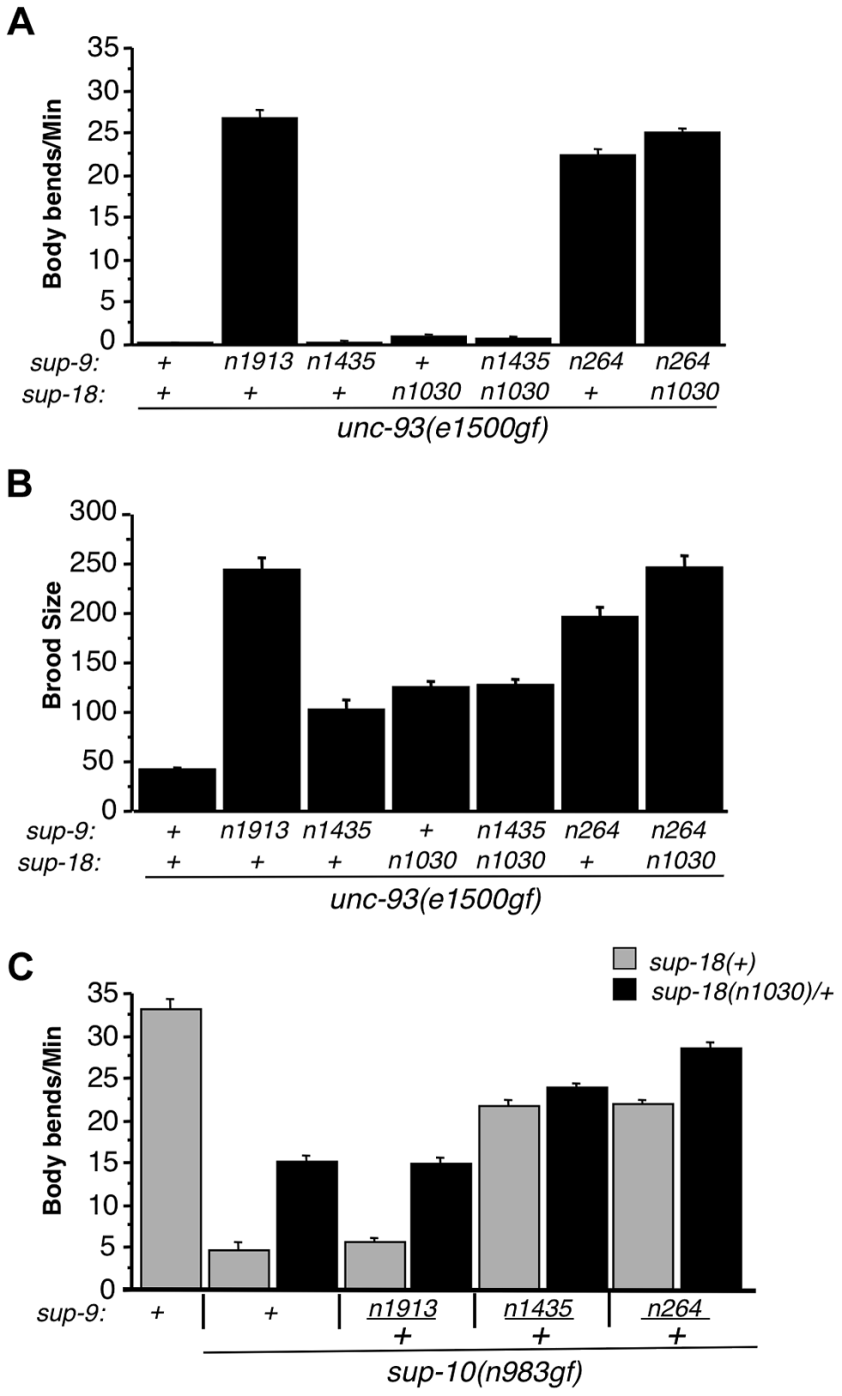
The *sup-9(n1435)* and *sup-18(lf)* mutations act similarly in suppressing rubberband Unc mutant phenotypes. (A) The effects of *sup-9(n1435)* and *sup-18(n1030)* mutations on the locomotion (body bends) of *unc-93(gf)* mutants are not additive. At least 12 young hermaphrodites were assayed for each genotype. Error bars, means ± SEMs. (B) The suppressive effects of *sup-9(n1435)* and *sup-18(n1030)* on the brood size defects of *unc-93(e1500gf)* mutants are not additive. Brood sizes of hermaphrodites were determined by picking L4 hermaphrodites to single plates, passaging them each day to a new plate and scoring the number of progeny on each plate. At least 11 animals were assayed for each genotype. Error bars, means ± SEMs. (C) Body bend assays of young males. At least 15 animals were assayed for each genotype. Error bars, means ± SEMs.

The similarity of *sup-18(lf)* mutations and *sup-9(n1435)* in preferentially suppressing *sup-10(n983gf)* defects compared to those of *unc-93(e1500gf)* mutants suggests that *sup-18(lf)* mutations and the *sup-9(n1435)* mutation might act via the same mechanism. If so, *n1435* might have no suppressive activity in the absence of *sup-18*. Indeed, the locomotory rate of the *sup-9(n1435); unc-93(e1500gf) sup-18(n1030)* triple mutant was similar to that of either the *sup-9(n1435); unc-93(e1500gf)* or the *unc-93(e1500gf) sup-18(n1030)* double mutant (Fig. 5A). This effect appears to be specific for *sup-9(n1435)*, as a different weak *sup-9* allele, *n264*, was enhanced by *sup-18(n1030)* (Fig. 5A). We also assayed the brood size of *unc-93(e1500gf)* mutants in the presence of either or both *sup-18(n1030)* and *sup-9(n1435)*. For example, although the low brood size of *unc-93(e1500gf)* mutants was restored to wild-type levels by the null mutation *sup-9(n1913)* (Fig. 5B), *sup-9(n1435)* and *sup-18(n1030)* single mutations or *sup-9(n1435); sup-18(n1030)* double mutations only partially rescued the brood size of *unc-93(e1500gf)* mutants and the double mutations acted similarly to the *sup-18(n1030)* single mutation (Fig. 5B). As was the case for locomotion, for brood size *sup-18(n1030)* enhanced the effect of the weak loss-of-function allele, *sup-9(n264)* on *unc-93(e1500gf)* mutants (Fig. 5B). The lack of an additive effect of *sup-18(n1030)* and *sup-9(n1435)* in suppressing the locomotion and brood size defects of *unc-93(e1500gf)* mutants suggests that *sup-9(n1435)* and *sup-18(n1030)* mutations likely act through the same pathway.

To further examine this hypothesis, we tested for an additive effect between *sup-18(n1030)/+* and *sup-9(n1435)/+* in their suppression of the locomotory defects of *sup-10(n983gf)* mutants. (A test for an additive effect of *sup-18(n1030)* and *sup-9(n1435)* homozygous mutations would not be informative, as both mutations fully suppress the locomotory defect of *sup-10(n983gf)* mutants.) We found that *sup-10(n983gf)* males heterozygous for either *sup-9(n1435)/+* or *sup-18(n1030)/+* are partially suppressed for the locomotory defects (Fig. 5C). The *sup-9(n1435)/+; sup-18(n1030)/+; sup-10(n983gf)* male triple mutant moved only slightly better than *sup-9(n1435)/+; sup-10(n983gf)* mutants (23.7±0.6 vs. 21.6±0.9, mean ± SEM, respectively) (Fig. 5C), suggesting a very weak additive effect of *sup-18(n1030)*/+ and *sup-9(n1435)*/+. [This small effect might be caused by the presence in these animals of wild-type SUP-9 dimers at a fourth the wild-type level; this SUP-9 would respond to *sup-18(n1030)/+* effects.] To verify the specificity of the interaction between *sup-18(n1030)* and *sup-9(n1435)*, we tested *sup-9(n264). sup-9(n264)/+* is as strong as *sup-9(n1435)/+* in suppressing the locomotory defects of *sup-10(n983gf)* mutants. However, unlike *sup-9(n1435)/+; sup-18(n1030)/+; sup-10(n983gf)* mutants, *sup-9(n264)/+; sup-18(n1030)/+; sup-10(n983gf)* mutants moved better than *sup-9(n264)/+; sup-10(n983gf)* mutants (28.5±0.5 vs. 21.3±0.6 bends/minute, mean ± SEM, respectively) (Fig. 5C). This result is consistent with the finding that *sup-18(n1030)* and *sup-9(n1435)* lack an obviously additive effect in suppressing the locomotion and egg-laying defects of *unc-93(e1500gf)* mutants (Fig. 5A and B) and supports our conclusion that *sup-9(n1435)* and *sup-18(lf)* alleles act in the same pathway in affecting rubberband Unc mutants.

### The *sup-9(n1435)* mutation affects a conserved region in the C-terminal domain of SUP-9

**Figure 6:**
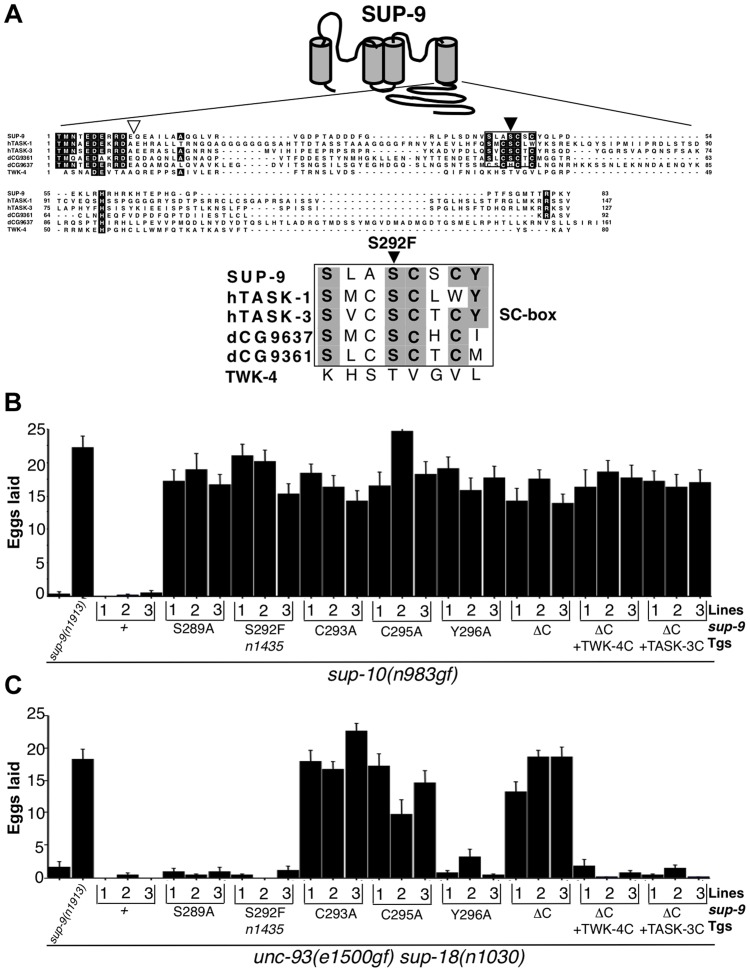
*sup-9(n1435)* is mutated in a conserved SC box at the C-terminus of two-pore domain K^+^ channels. (A) Top, schematic diagram of the proposed structure of the SUP-9 channel. Cylinders represent the four presumptive transmembrane domains. Middle, alignment of the C-terminal cytoplasmic tail of SUP-9 (amino acids 247–329) and related channels: human TASK-1 (aa 248–394) Genebank Acc#NP_002237; human TASK-3 (aa 248–374) Genebank Acc#Q9NPC2; *Drosophila* predicted protein CG9637 (aa 238–398) Genebank Acc# AAF54970; *Drosophila* predicted protein CG9361 (aa 249–340) Genebank Acc# AAF54374; *C. elegans* TWK-4 (aa 255–334) Genebank Acc#AAC32857. Residues conserved among at least four of the channels are shaded. Box, SC box; black triangle, site of the *n1435* mutation; white triangle, site of SUP-9::TWK-4 C-terminal fusion. Bottom, alignment of SC boxes from SUP-9 and related channels. Residues conserved among SUP-9 and at least two other channels are shaded. (B, C) Mutant animals of the indicated genotypes were assayed for egg-laying rate. Young adult hermaphrodites were allowed to lay eggs for 3 hrs on a bacterial lawn, and eggs or larvae on the plate were counted. Independently derived transgenic lines of (B) *sup-10(n983gf) lin-15* or (C) *unc-93(e1500gf) sup-18(n1030); lin-15* animals containing both a *lin-15*-rescuing transgene and the P*_myo-3_ sup-9* derivatives are indicated. Error bars, means ± SEMs of at least 14 animals.

We determined the *sup-9* coding sequences in *sup-9(n1435)* mutants and identified a C-to-T transition within codon 292, leading to a serine-to-phenylalanine substitution within the predicted intracellular C-terminal domain of SUP-9 (Fig. 6A). Although SUP-9 is 41%–47% identical in amino acid sequence over its entire region to several TASK-family two-pore domain K^+^ channels [28], the C-terminal cytoplasmic domain of SUP-9 is poorly conserved among these channels (Fig. 6A). However, the serine affected by the *n1435* mutation is located in a small conserved stretch of amino acids with the sequence SxxSCxCY (Fig. 6A). We named this region the SC (Serine-Cysteine-rich)-box. The residues in the SC-box do not correspond to any reported motifs, including phosphorylation sites, as defined by the protein motif database PROSITE [47]. Variations of the SC-box are found in the human TASK-1 and TASK-3 channels and in two *Drosophila* two-pore domain K^+^ channels (Fig. 6A). We have not found an SC Box in other human two-pore domain K^+^ channels (I. de la Cruz and H. R. Horvitz, unpublished observations) or in TWK-4 (C40C9.1), a *C. elegans* two-pore domain K^+^ channel that is 41% identical to and the most closely related *C. elegans* channel to SUP-9 (Fig. 6A).

To determine if other residues in the SC-box of SUP-9 might function like the S292F substitution, we performed an *in vivo* mutagenesis study of the SC-box. We mutated residues S289, C293, C295 and Y296 to alanine individually and compared their effects in suppressing the egg-laying defects of the *sup-10(n983gf)* and *unc-93(e1500gf) sup-18(n1030)* double mutants. When assayed over a 3 hr period, both mutant strains laid fewer than three eggs, and a *sup-9(n1913)* null mutation drastically increased egg laying by both strains (Fig. 6B, C). As a control, overexpression of a *sup-9(+)* cDNA driven by the *myo-3* promoter (P*_myo-3_ sup-9(+)*) in either *sup-10(n983gf)* or *unc-93(e1500gf) sup-18(n1030)* mutants did not increase egg-laying in each of three independent transgenic lines. By contrast, overexpression of a *sup-9* cDNA containing the *n1435* mutation (P*_myo-3_ sup-9(n1435)*) dominantly suppressed the egg-laying defects of *sup-10(n983gf)* mutants (Fig. 6B) but not those of *unc-93(e1500gf) sup-18(n1030)* animals (Fig. 6C). These results establish an *in vivo* assay for identifying mutations in *sup-9* that preferentially suppress *sup-10(n983gf)* over *unc-93(e1500gf)* mutations.

A P*_myo-3_ sup-9(S289A)* and a P*_myo-3_ sup-9(Y296A)* transgene suppressed the defects of *sup-10(n983gf)* mutants but not of *unc-93(e1500gf) sup-18(n1030)* mutants, suggesting that the *S289A* and *Y296A* mutations act similarly to *n1435* to mediate the gene-specific effects of *sup-18(lf)* mutations. By contrast, the cysteine-to-alanine mutations at residues 293 and 295 of SUP-9 suppressed both *sup-10(n983gf)* and *unc-93(e1500gf) sup-18(n1030)* mutants (Fig. 6B, C). We suggest that these mutations when overexpressed have a dominant-negative effect on the wild-type *sup-9* allele.

To further understand how its C-terminal domain affects SUP-9 activity, we deleted in the *sup-9* cDNA the region encoding the SUP-9 C-terminal cytoplasmic domain. We also replaced this region with the corresponding region of *twk-4*, which encodes a two-pore domain K^+^ channel without an SC-box, or of TASK-3, a mammalian homolog that contains an SC-box (Fig. 6). Deletion of the SUP-9 C-terminal domain caused suppression of both the *sup-10(n983gf)* and *unc-93(e1500gf) sup-18(n1030)* mutant phenotypes, suggesting that the truncated form of SUP-9 acts as a dominant-negative protein. Interestingly, both the *sup-9::twk-4* and *sup-9::TASK-3* fusion transgenes suppressed the *sup-10(n983gf)* egg-laying defect (Fig. 6B) but failed to suppress that of the *unc-93(e1500gf) sup-18(n1030)* mutants (Fig. 6C), suggesting that these fusion transgenes act similarly to *sup-9(n1435)* and affect rubberband Unc mutants in a gene-specific manner.

**Figure 7:**
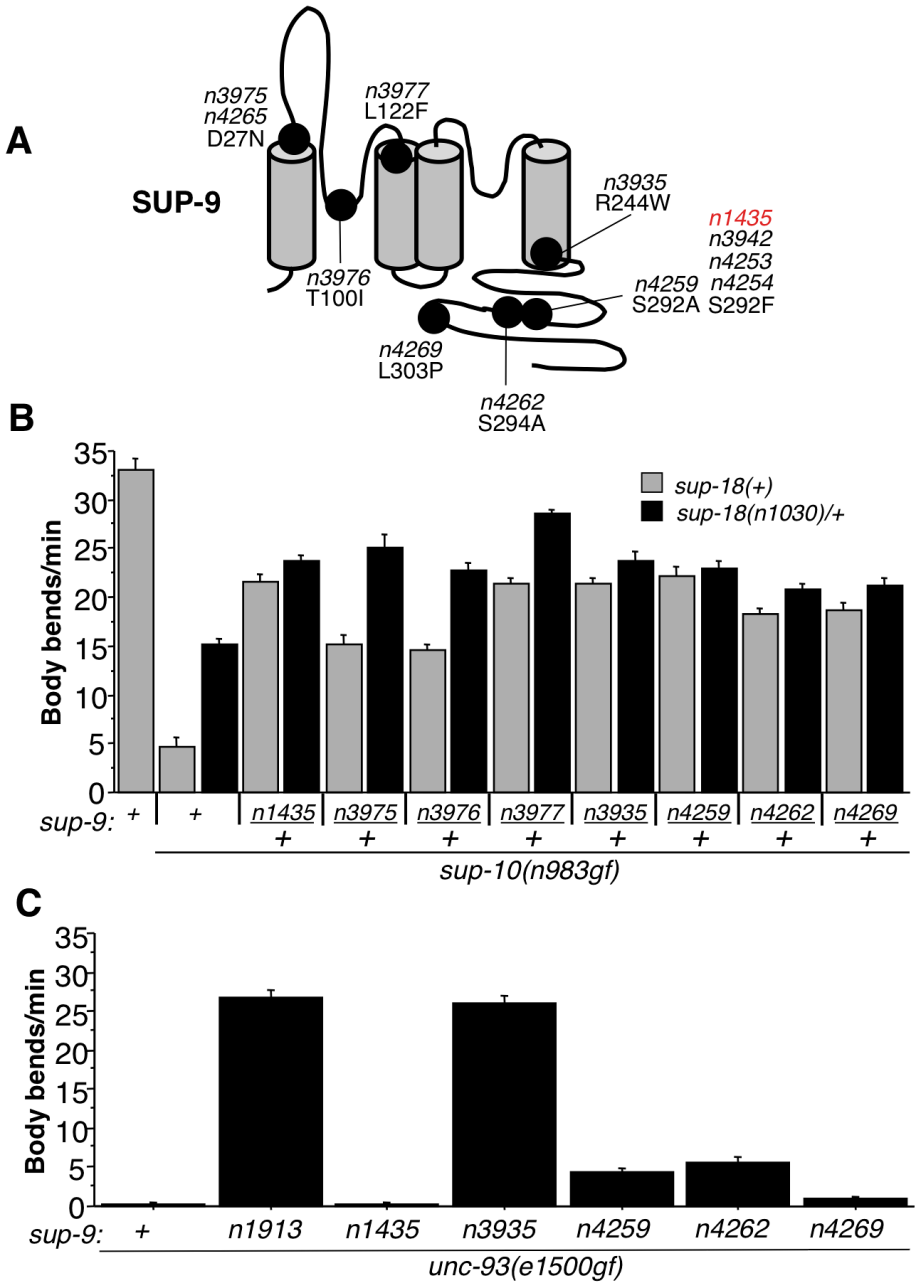
Characterization of novel *sup-9* alleles. (A) Schematic of proposed SUP-9 functional domains and identified missense mutations. (B) Effects of various combinations of *sup-9* and *sup-18* alleles on the locomotion of *sup-10(n983gf)* animals. (C) Effects of *sup-9* alleles on the locomotion of *unc-93(e1500gf)* animals.

To identify more *sup-9* mutations that act similarly to *sup-9(n1435)*, we performed a genetic screen for mutations that semidominantly suppressed the *sup-10(n983gf)* rubberband phenotype (see Materials and Methods). We isolated eight mutations of *sup-9* that define seven novel alleles (*n3975* (*n4265*), *n3976*, *n3977*, *n3935*, *n4259*, *n4262* and *n4269*) (Fig. 7A) and three additional mutations (*n3942*, *n4253*, *n4254*) that contained the same C-to-T transition and therefore caused the same S292F substitution as *sup-9(n1435)*. As heterozygotes, five of the seven novel alleles (*n3977*, *n3935*, *n4259*, *n4262*, *n4269*) were stronger suppressors of *sup-10(n983gf)* mutants like *sup-9(n1435)/+* (∼23 bends/minute), while the other two (*n3975*, *n3976*) were weaker (Fig. 7B). These mutations affect six different regions of SUP-9 (Fig. 7A), including the first (*n3975*) and second (*n3977*) transmembrane domains, the first pore domain (*n3976*), the beginning of the C-terminal cytoplasmic domain (*n3935*), the SC-box (*n4259* and *n4262*), and a region C-terminal to the SC-box (*n4269*)

To determine if these novel *sup-9* mutations conferred resistance to *sup-18* activation or if they were simply dominant-negative lf mutations, we tested their responsiveness to changes in *sup-18* levels in a similar manner to that used for testing *sup-9(n1435)* (Table 2 and Fig. 5). By comparing the locomotion of *sup-9(mut)/+; sup-18(n1030)/+; sup-10(n983gf)* mutants to that of *sup-18(n1030)/+; sup-10(n983gf)* mutants, we found that *sup-9(n3935)/+*, *sup-9(n4259)/+*, *sup-9(n4262)/+* and *sup-9(n4269)/+* caused a weak effect similar to that by *sup-9(n1435)/+*, while *n3975/+*, *n3976/+* and *n3977/+* caused a significant improvement in locomotory rate in response to a change in *sup-18* levels (Fig. 7B). This result suggests that the channels generated by the three mutations *n3975*, *n3976* and *n3977* have impaired ability to generate K^+^ currents but retain regulation by SUP-18.

In addition to its *sup-18* insensitivity, *sup-9(n1435)* was also a weak suppressor of the *unc-93(e1500gf)* locomotory defect, while the null mutation *sup-9(n1913)* completely suppressed the *unc-93(gf)* defect (Tables 1 and 5). Similarly, *sup-9(n4259)*, *sup-9(n4262)* and *sup-9(n4269)* only weakly suppressed the locomotory defects of *unc-93(e1500gf)* animals (Fig. 7C), suggesting that these mutations belong to the class of *sup-9* alleles defined by *sup-9(n1435)*. However, *sup-9(n3935)* completely suppressed the locomotory defects of *unc-93(e1500gf)* animals (Fig. 7C), indicating that *sup-9(n3935)* was not only insensitive to *sup-18* but also resistant to the activating effects of *unc-93(e1500gf)*. Thus, mutations affecting different residues of SUP-9 confer differential channel sensitivity to its regulatory subunits.

## Discussion

### *sup-18* encodes a transmembrane protein orthologous to mammalian iodotyrosine deiodinase

Two-pore domain K^+^ channels are widely expressed and play important roles in regulating resting membrane potentials of cells [15], [17]. However, very little is known about protein factors with which these channels interact. We previously identified UNC-93 and SUP-10 as presumptive regulatory subunits of the SUP-9 two-pore domain K^+^ channel. We now suggest that SUP-18 also regulates the SUP-9/UNC-93/SUP-10 channel complex.

*sup-18* encodes the *C. elegans* ortholog of mammalian iodotyrosine deiodinase (IYD), which belongs to the NADH oxidase/flavin reductase superfamily [7], [8]. By oxidizing NADH using flavin mononucleotide (FMN) as a cofactor, IYD catalyzes the recycling of iodide from monoiodotyrosine and diiodotyrosine, two major byproducts in the synthesis of thyroid hormones [7], [8]. Lack of IYD function can lead to congenital hypothyroidism [12], [13]. In *C. elegans*, no SUP-18 function besides regulating the SUP-9 channel has been identified. The enzymatic activity of SUP-18 remains to be defined.

Little is known about the metabolism and function of iodide in nematodes. The *C. elegans* genome contains two genes, *ZK822.5* and *F52H2.4*, that encode homologs of the mammalian sodium/iodide symporter, which enriches iodide in the thyroid cells by active membrane transport [48]. The presence of both SUP-18 IYD and sodium/iodide symporter-like proteins suggests that iodide functions biologically in *C. elegans*. Although iodide appears not to be an essential trace element in the culture medium of *C. elegans* [49], it is possible that residual iodide in components of that medium can provide sufficient nutritional support for survival. *C. elegans* lacks homologs of mammalian iodothyronine deiodinase (I. de la Cruz, L. Ma and H. R. Horvitz, unpublished observations), enzymes that remove the iodine moieties from the precursor thyroxine (T4) and generate the more potent thyroid hormone 3, 5, 3′-triiodothyronine [50], which suggests that thyroid hormones might not be synthesized in *C. elegans*.

IYDs across metazoan species share a similar enzymatic activity in reductive deiodination of diiodotyrosine [51], and it seems likely that SUP-18 acts similarly in *C. elegans*. Like mammalian IYDs, SUP-18 contains a presumptive N-terminal transmembrane domain that is required for full activity. Interestingly, the SUP-18 intracellular region lacking the transmembrane domain could still partially activate the SUP-9 channel, suggesting that membrane association is not absolutely required for SUP-9 activation by SUP-18. Membrane association is important for mammalian IYD enzymatic activities [5], [52], [53].

The presence of a transmembrane domain suggests that SUP-18 IYD might interact with other transmembrane proteins. The genetic interactions we observe between *sup-18* and the genes that encode the SUP-9/UNC-93/SUP-10 two-pore domain K^+^ channel complex support this hypothesis. Based on expression studies, we conclude that SUP-18 and SUP-10 localize to similar subcellular structures within muscle cells, further supporting the idea that SUP-18 and the channel complex interact physically. We found that transgenic expression of the SUP-18 intracellular domain could enhance the expression of the rubberband phenotype, suggesting that plasma membrane localization is not essential for SUP-18 function. Nonetheless, the expression of the full-length SUP-18 was more potent than the expression of the SUP-18 intracellular domain in rescuing the rubberband Unc phenotypes of *sup-18(lf); sup-10(n983gf)* mutants, suggesting that the presence of a transmembrane domain in SUP-18 IYD could enhance the activity of SUP-18 by targeting SUP-18 to the plasma membrane.

The crystal structure of mouse IYD reveals that eight residues contact the FMN cofactor: R96, R97, S98, R100, P123, S124, T235 and R275 [54]. Except T235, which is replaced by a serine in SUP-18, these residues are completely conserved (Figure 1C, yellow boxes). Furthermore, the *sup-18(n1010)* missense mutation leads to an S137N substitution at the position equivalent to the mouse S98 residue, likely disrupting the binding of FMN. This high degree of conservation at the cofactor binding site suggests that SUP-18 likely retains the ability to bind FMN and likely has a catalytic activity.

Three IYD missense mutations that cause hypothyroidism (R101W, I116T, and A220T) affect residues that are conserved in SUP-18 [12], [55] (Fig. 1C, red boxes). A fourth human mutation replaces F105 and I106 with a leucine [8]. The phenylalanine at position 105 is conserved in SUP-18 (Fig. 1C). The conservation of residues associated with IYD function supports the hypothesis that SUP-18 regulates the SUP-9 two-pore domain K^+^ channel complex via an enzymatic activity. The SUP-18 substrate remains to be elucidated.

That SUP-18 might function as a NADH oxidase/flavin reductase raises the intriguing possibility that SUP-18 might couple the metabolic state of muscle cells with membrane excitability. Mammalian K_v_β voltage-gated K^+^ channel regulatory subunits [56], which belong to the aldo-keto reductase superfamily [57], [58], have similarly been proposed to couple metabolic state with cell excitability based on indirect evidence. K_v_β2 has an NADP^+^ cofactor bound in its active site and a catalytic triad spaced appropriately to engage in enzymatic activity [58]. Although suggestive of an enzymatic activity, no substrate has been reported for K_v_β subunits. While K_v_β2 knockout mice have seizures and reduced lifespans, mice carrying a catalytic null mutation in K_v_β2 have a wild-type phenotype, suggesting that if an enzymatic activity for K_v_β2 exists, it is functionally dispensable *in vivo* [59]. By contrast, the predicted catalytic mutation *sup-18(n1010)* behaves like a null mutation in its inability to activate the SUP-9 channel, even though the SUP-18(n1010) protein is synthesized and localized normally to the cell surface of muscle cells (I. de la Cruz and H. R. Horvitz, unpublished observations). Five other *sup-18* mutations affecting highly conserved residues in the NADH oxidase/flavin reductase domain also behave like null mutations, consistent with the hypothesis that SUP-18 enzymatic activity is essential for its function.

### *sup-18(lf)* mutations define a new class of gene-specific suppressors of the rubberband Unc mutants

*sup-18(lf)* mutations strongly suppress *sup-10(n983gf)* mutants and weakly suppress *unc-93(e1500gf)* mutants. Certain specific mutations of *sup-9*, including *n1435*, *n4259*, *n4262*, and *n4269*, act similarly to *sup-18(lf)* and are strong suppressors of *sup-10(n983gf)* mutants and weak suppressors of *unc-93(e1500gf)* mutants. Together these *sup-9* mutations and *sup-18(lf)* mutations represent a novel class of mutations that exhibit gene-specific suppression of the rubberband Unc mutants and are distinct from another class of gene-specific suppressors we identified previously, mutations in three splicing factor genes that strongly suppress *unc-93(e1500gf)* and *sup-10(n983gf)* but do not obviously suppress *unc-93(n200gf)* or *sup-9(n1550gf)* [60]–[62]. The difference between *sup-18(lf)* and *sup-9(n1435*, *n4259*, *n4262*, *n4269)* mutations and the splicing factor mutations in their patterns of suppressing the rubberband Unc mutants suggests that these two classes of suppressors function by distinct mechanisms.

### SUP-18 is an activator of the SUP-9 two-pore domain K^+^ channel

SUP-9 is closely related to the subfamily of two-pore domain K^+^ channels that include human TASK-1 and TASK-3 [28]. TASK-1 is activated by multiple factors, including extracellular pH [22], [23], [63], inhalational anesthetics such as halothane [24] and oxygen [64]. TASK-1 is directly inhibited by sub-micromolar levels of the cannabinoid neurotransmitter anandamide [65] and by neuromodulators such as thyrotropin releasing hormone (TRH) [27]. A histidine residue in the first P-domain of TASK-1 modulates its sensitivity to pH [66], while a six amino acid stretch following its fourth transmembrane domain is required for both halothane activation and TRH suppression [24], [67]. Deletion of the TASK intracellular C-terminal domain, which contains the SC-box, does not change its basal activity or activation by halothane [24], [67], suggesting that the TASK-1 C-terminal domain and probably the SC-box represent an activation region that is required by some types of channel activator (*e.g.*, human IYD) but not by others (*e.g.*, halothane and pH). It remains to be determined whether IYD is involved in the inhibition of TASK-1 channel activity by TRH.

From our genetic analysis of the *sup-9(n1435)* mutation and site-directed mutagenesis of *sup-9*, we have defined the SC-box, a domain of SUP-9 required for SUP-10(n983gf)-specific activation. The importance of the SC-box in mediating this activation is supported by the results of a genetic screen in which we isolated additional *sup-9* mutations (Fig. 7) that act like *sup-9(n1435)* and cause distinct amino acid changes in (*n4259* (S292A), *n4262* (S294A)) or near (*n4269* (L303P)) the SC-box. Although conserved in the human TASK-1 and TASK-3 channels (Fig. 6A), no function has yet been assigned to the SC-box. Our analyses suggest that the SC-box and the C-terminal domain of SUP-9 likely mediate the functional interaction between SUP-9 and SUP-10/SUP-18 but are dispensable for interaction with UNC-93. We found that replacing the C-terminal domain of SUP-9 with the corresponding region of TWK-4 (which lacks an SC-box) or of TASK-3 (with an SC-box) makes the fusion channels behave like SUP-9(n1435), consistent with the model that the SC-box is required for SUP-9 activation by SUP-10(n983gf) and SUP-18 (based on the TWK-4 data) and suggests that SC-box-dependent activation requires one or more nearby residues in the C-terminal domain (based on the TASK-3 data). The *unc-93(e1500gf)* mutation results in a glycine-to-arginine substitution at amino acid 388 in one of the putative transmembrane domains [33], suggesting that the UNC-93(gf) protein activates SUP-9 through an interaction involving transmembrane domains, without a need for the SC-box or the rest of the cytoplasmic domain.

We describe three important properties of the unusual *sup-9(n1435)* mutation. First, SUP-9(n1435) channels cannot be activated by SUP-10(n983gf). Second, SUP-9(n1435) channels are insensitive to SUP-18 activity. Third, SUP-9(n1435) channels can be activated by UNC-93(e1500gf). The existence of a channel mutation that is insensitive to both SUP-18 and SUP-10(n983gf) suggests that these two inputs act through a common pathway. A mutant channel that can be activated by UNC-93(e1500gf) but not by SUP-10(n983gf) suggests that there is an independent pathway for SUP-9 activation by UNC-93.

**Figure 8:**
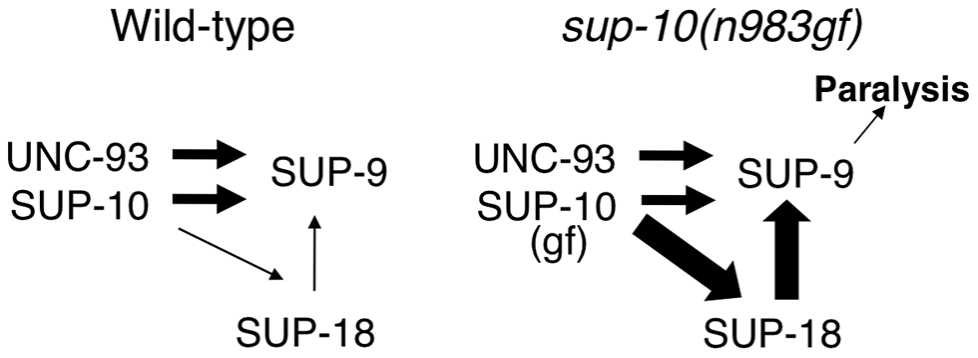
A model for activation of the SUP-9 channel by multiple subunits. In this model, SUP-10 and UNC-93 act independently of SUP-18 to activate SUP-9. In addition, SUP-10 enhances SUP-18, which activates SUP-9 through a distinct pathway. SUP-10(n983gf) over-enhances SUP-18, which over-activates SUP-9 and leads to paralysis. The widths of the arrows pointing at SUP-9 are representative of their relative strengths in sustaining gf activity, with thicker arrows representing a larger contribution.

We propose a model to explain the functional interactions between SUP-18 and SUP-9/UNC-93/SUP-10 (Fig. 8). In this model, SUP-10 and UNC-93 have an essential role in and are both required for activating SUP-9 channel, since the *n1550* gf mutation in *sup-9* is completely suppressed by *sup-10(lf)* and *unc-93(lf)* mutations [38]. SUP-18 activates SUP-9 only weakly and relies on SUP-10 for this activation (Fig. 8). SUP-10(n983gf) enhances the activity of SUP-18 and results in over-activation of SUP-9 by SUP-18. Our model is consistent with the genetic and molecular evidence described in this and previous studies [28]–[31], [33] and should provide a framework for understanding the interactions of SUP-18 and the SUP-9/UNC-93/SUP-10 channel complex. Our results do not distinguish whether SUP-18 regulates the SUP-9/UNC-93/SUP-10 complex via a direct physical interaction or indirectly through an unknown factor or factors.

In short, we identified SUP-18 IYD as a functional regulator of the SUP-9/UNC-93/SUP-10 two-pore domain K^+^ channel complex. We also defined an evolutionarily conserved serine-cysteine-rich domain, the SC-box, in the C-terminal region of SUP-9 and showed that this region is required for activation of the channel by SUP-18. Since IYD is likely to be an NADH oxidase/flavin reductase that uses the ubiquitous energy carrier molecule NADH as a coenzyme, our study suggests that IYD might couple cellular metabolic state to two-pore domain K^+^ channel activities. Future molecular analyses should reveal the mechanism underlying the interaction between the SUP-9 two-pore domain K^+^ channels and SUP-18 IYD.

## Materials and Methods

### Strains and genetics

*C. elegans* strains were cultured as described [49], except that *E. coli* strain HB101 was used instead of OP50 as a food source. Strains were grown at 20°C unless otherwise noted. The following mutations were used in this study:

LGII *sup-9(n213*, *n233*, *n264* [31], *n1016*, *n1025* [30], *n1435*, *n1550gf* [29], *lr35*, *lr38*, *lr45*, *lr100*, *lr129*, *lr142*, *n1472*, *n1557*, *n1913* [28], *n3935*, *n3942*, *n3975*, *n3976*, *n3977*, *n4253*, *n4254*, *n4259*, *n4262*, *n4265*, *n4269* (this study)).

LGIII *unc-93(e1500gf*, *n200gf)* [31], *sma-3(e491)* [49], *mec-14(u55)* [68], *ncl-1(e1865)* [69], *unc-36(e251)* [49]. *sup-18(n463*, *n527*, *n528*, *n1010*, *n1014*, *n1015*, *n1022*, *n1030*, *n1033*, *n1035*, *n1036* [30], *n1038*, *n1471*, *n1539*, *n1548*, *n1554*, *n1556*, *n1558* (this study)).

LGX *sup-10(n183* [31], *n1008*, *n983gf* [30], *n4025*, *n4026* (this study)), *lin-15(n765ts)* [70].

### Isolation of partially suppressed *unc-93(e1500gf)* mutants

Since lf mutations in *sup-10* completely suppress the paralysis of *unc-93(e1500gf)* mutants [31], we reasoned that partial lf mutations of *sup-10* would partially suppress the *unc-93(e1500gf)* locomotory phenotype. To isolate such partial lf *sup-10* mutations, we performed an EMS F_2_ genetic screen for partial suppressors of the locomotory defects of *unc-93(e1500gf)* mutants. From 17,500 haploid genomes screened, we isolated over 30 strong suppressors and seven weak suppressors. We assigned two of the seven weak suppressors, *n4025* and *n4026*, to the *sup-10* locus by complementation tests and three others to the *unc-93* locus. All seven were saved for future analyses.

### Mapping and cloning of *sup-18*

34 Sma non-Unc and 23 Unc non-Sma progeny were isolated from a *sma-3 mec-14 ncl-1 unc-36/sup-18* parent. Scoring of the *ncl-1* and *sup-18* phenotypes identified the 57 recombination events to be distributed in the three relevant intervals as follows: *sma-3* (30/57) *ncl-1* (3/57) *sup-18* (24/57) *unc-36*. A pool of cosmids C33C3, C08C3, C27D11, C02C2, C39F10 and C44C9 at 1 ng/µL each and a *rol-6* marker [71] at 80 ng/µL were injected into *sup-18(n1010); sup-10(n983gf)* animals. Two Rol transgenic lines were obtained, one of which generated rubberband Unc animals. The four middle cosmids were injected separately, and C02C2 yielded 5/5 rescued lines, while transgenes containing cosmids C08C3 (0/7), C27D11 (0/5) or C39F10 (0/9) showed no rescue.

RT-PCR was performed on cDNA from the wild-type N2 strain using the primers 5′-TTGAAAACCCCTGTTAAATAC-3′ and 5′-CGAGTTTCTAATAAAAATAAACC-3′. PCR products were cloned into pBSKII (Stratagene), and their sequences determined. 5′ and 3′ RACE were performed using the corresponding kits (Gibco).

### Molecular biology

Genomic subclones of cosmid C02C2 were generated in pBSKII (Stratagene). The subclones, in the order shown in Figure 1, spanned the following sequences (Genebank Acc#L23649): *Eco*RV (9,790) - *Eco*RV (21,098); *Pst*I (23,699) - *Pst*I (32,833); *Pst*I (23,699) - *Sac*I (28,185); *Bst*BI (24,448)-*Sac*I (28,185); and *Hind*III (24,671) - *Hind*III (27,169).

All PCR amplifications used in plasmid constructions were performed using *Pfu* polymerase, and the sequences of their products were determined. The P*_myo-3_ sup-18* vectors for ectopic expression of wild-type or mutant *sup-18* alleles were generated by PCR amplification of the respective coding regions from *sup-18* cDNAs using primers that introduced *Nhe*I and *Sac*I sites at the 5′ and 3′ ends, respectively, and cloned into vector pPD95.86 (from A. Fire). P*_myo-3_ sup-18(intra)* was similarly constructed, except that the 5′ primer began at codon 66 of *sup-18*. The *gfp*-tagged version of this vector was created by PCR amplification of the *gfp* coding sequence from vector pPD95.77 (from A. Fire) and subcloned into P*_myo-3_ sup-18(intra)* just prior to the start codon of the *sup-18* sequence.

P*_myo-3_ mIYD* (mouse *IYD*) was generated by PCR amplification of the coding region of the mouse cDNA (Gene Bank AK002363) with 5′ and 3′ primers containing *Nhe*I and *Eco*RV sites, respectively, and subcloning the PCR products into pPD95.86 at the *Nhe*I and *Sac*I (blunted) sites. P*_myo-3_ mIYD::gfp* was generated by a similar strategy using a 5′ primer containing an *Nhe*I site and a 3′ primer that did not include the stop codon of *mIYD* but instead contained a *Bam*HI site. The *myo-3* promoter from pPD95.86 was subcloned into pPD95.77, such that upon subcloning of the *mIYD* PCR fragment into the *Nhe*I and *Bam*HI sites of the vector the *myo-3* promoter drove expression of the *mIYD* gene fused in-frame at its 3′ end to *gfp*.

The *sup-18::gfp* genomic fusion was constructed by introducing *Sph*I sites at the ends of a *gfp* cassette by PCR amplification of plasmid pPD95.77 (from A. Fire) and subsequent subcloning into the single *Sph*I site contained within a 9.1 kb *Pst*I genomic *sup-18* rescuing fragment. The resulting fusion contained 6.5 kb of promoter sequence, the entire *sup-18* coding region with *gfp* inserted between the transmembrane and NADH oxidase/flavin reductase domains and 1.1 kb of 3′ UTR and downstream sequence.

The *sup-10::gfp* fusion used in colocalization studies was constructed by subcloning a 7.3 kb *Mfe*I genomic fragment from cosmid C27G6 containing *sup-10* into the *Eco*RI site of pBSKII. A 6.4 kb *Pst* I fragment was subcloned from this vector into pPD95.77, which contained 3.5 kb of promoter sequence and the *sup-10* coding region. Using PCR, we introduced a *Sal*I site immediately preceding the stop codon of *sup-10* to create an in-frame fusion with the *gfp* coding sequence.

*sup-18::β-galactosidase* fusions were created by PCR amplification of 1869 bp of 5′ *sup-18* promoter sequence and subcloning the product into the *Sph*I and *Pst*I sites of pPD34.110 (from A. Fire) to generate P*_sup-18_ TM-β-Gal*, which contains a synthetic transmembrane sequence [45] followed by the β-galactosidase coding sequence [72]. *sup-18* genomic coding sequence spanning codons 1–42, 1–70 and 1–301 were PCR-amplified from the minimal rescuing fragment with 5′ and 3′ primers that contained *Pst*I and *Bam*HI sites, respectively, and subcloned into these sites in P*_sup-18_ TM-β-Gal*. The synthetic transmembrane domain was deleted from these plasmids by excising the *Kpn*I fragment containing this domain. A signal sequence [73] was inserted into these vectors using standard PCR techniques.

The *GST::sup-18(N)* and *MBP::sup-18(N)* fusion genes used to generate and purify anti-SUP-18 antibodies were generated by PCR amplification of codons 1–258 of the *sup-18* cDNA and subcloning the products into pGEX-2T (Pharmacia) and pMal-2c (NEB) vectors.

The full-length *twk-4* cDNA was cloned by RT-PCR with primers 5′-CTCTGCTAGCAATGCATCAAATTGACGGAAAATCTGC-3′ and 5′-AGAGGATCCATATAGTTCAAGATCCACCAGATG-3′ from wild-type mixed-stage RNA. The sequence of the *twk-4* cDNA obtained was in agreement with its predicted sequence (GenBank Acc#AF083646). The C-terminal cytoplasmic domain of *sup-9* from the P*_myo-3_ sup-9* vector (codons 257–329 of *sup-9*) was replaced by *twk-4* codons (265–365) using standard PCR ligation techniques to generate P*_myo-3_ sup-9::twk-4*. Site-directed mutagenesis of the SC-box in the P*_myo-3_ sup-9* vector was likewise performed.

### Body-bend assay

Young adults were individually picked to plates with HB101 bacteria, and body-bends were counted for one minute using a dissecting microscope as described [74].

### Antibody and immunostaining

A GST::SUP-18(N) fusion protein was expressed in *E. coli* and the insoluble protein was purified by SDS-PAGE and used to immunize rabbits. Antisera were purified by binding to the MBP::SUP-18 protein immobilized on nitrocellulose strips and elution with 100 mM glycine-HCl (pH 2.5). This antibody could detect SUP-18 overexpressed in the body-wall muscles (Fig. 2H) but failed to detect endogenous SUP-18.

For immunofluorescence experiments, worms at mixed stages were fixed in 1% paraformaldehyde for 2 hrs at 4°C and permeabilized as described [75]. For colocalization studies, transgenic worms were stained with primary antibodies at 1∶200 dilution and a secondary goat-anti-rabbit antibody conjugated with Texas Red (Jackson Labs). Worms were viewed using confocal microscopy.

### Transgenic animals

Germline transformation experiments were performed using standard methods [71]. Transgenic strains carrying the *lin-15(n765ts)* mutation contained the coinjection marker pL15EK(*lin-15(+)*) at 50 ng/µL [70], and transgenic animals were identified by their non-Muv phenotype at 22.5°C. The dominant *rol-6* plasmid [71] was used at 100 ng/µl during cosmid rescue experiments, and transgenic animals were identified by their Rol phenotype. The dominant *myo-3::gfp* fusion vector pPD93.97 (from A. Fire) was used where indicated at 80 ng/µl, and transgenic animals were identified by GFP fluorescence. Experimental DNA was injected at 30–50 ng/µl.

### Isolation of novel *sup-9* alleles

One plausible genetic strategy for isolating *sup-9* alleles similar to *sup-9(n1435)* would be to perform an F_2_ screen for suppressors of the *sup-10(n983gf)* locomotory defect and then test these suppressors for their effects on the locomotory defect of *unc-93(e1500gf)* mutants. Most *sup-9* alleles isolated from such a screen would be typical lf alleles rather than rare alleles that would result in a SUP-9 protein specifically impaired in activation by SUP-10(gf) and SUP-18(+). We therefore opted for an alternative strategy based on the semidominance of the *sup-9(n1435)* mutation. While *sup-9* null mutations, such as *n1913*, recessively suppress the locomotory defects of *sup-10(n983gf)* mutants, *sup-9(n1435)* caused a strong semidominant suppression (Fig. 5C). As two-pore domain K^+^ channels are homodimers [66], [76], this semidominance likely reflects the formation of nonfunctional heterodimers composed of *n1435* and wild-type SUP-9 proteins. The strength of this semidominance (∼23 vs. ∼5 bends/minute for *sup-9(n1435)/+*; *sup-10(n983gf)* vs. *sup-10(n983gf)* mutants, respectively) formed the basis of an F_1_ screen for suppressors of the *sup-10(n983gf)* locomotory defect.

*sup-10(n983gf)* L4 hermaphrodites were mutagenized with EMS, and approximately 550,000 F1 progeny (1.1×10^6^ genomes) were screened for improved locomotion on agar plates. From 89 candidate suppressors, 35 mutants retested in the next generation, representing at least 31 independent isolates. To quantify the semidominant character of these mutants (*sup(new)*), wild-type males were crossed with homozygous mutant hermaphrodites to generate *sup(new)/+; sup-10(n983gf)/0* males, and their locomotory rate was scored. Because *sup-10* is on the X chromosome, this strategy generates males hemizygous for *sup-10(n983gf)* while heterozygous for autosomal mutations, providing a convenient assay of semidominance. Four mutations completely suppressed the rubberband Unc phenotype of males, with locomotory rates very similar to that of wild-type animals (∼33 bends/minute). We reasoned that these four mutants were likely lf alleles of *sup-10*, as such animals would be hemizygous for *sup-10*. We confirmed this assignment by determining the sequences of the *sup-10* locus and found mutations in all four strains (I. de la Cruz and H. R. Horvitz, unpublished observations). For the remaining strong mutants, we performed complementation tests with *sup-9*, *sup-18* and *unc-93* strains and identified 11 semidominant alleles of *sup-9* (see Results).
